# Prospective Multicenter Registry to Investigate the Clinical Feasibility of Combination Workflow With 90 W/4 s and Ablation Index‐Guided 50 W Ablation (PRECISE—COMBO 90 W/50 W Study)

**DOI:** 10.1002/joa3.70199

**Published:** 2025-09-26

**Authors:** Yuji Saito, Ryuta Watanabe, Koichi Nagashima, Yuji Wakamatsu, Shu Hirata, Moyuru Hirata, Masaomi Kimura, Junjiroh Koyama, Hideharu Okamatsu, Yuki Komatsu, Kenichi Hiroshima, Kaoru Tanno, Takahiro Furuya, Naoki Aizawa, Yuichiro Sakamoto, Taishi Kuwahara, Toshio Makita, Kenta Takahashi, Shiro Nakahara, Hirotsugu Sato, Hideyuki Aoki, Masahide Harada, Yuji Motoike, Jin Teranishi, Shin Takahara, Kenta Murotani, Yasuo Okumura

**Affiliations:** ^1^ Division of Cardiology, Department of Medicine Nihon University School of Medicine Tokyo Japan; ^2^ Department of Advanced Management of Cardiac Arrhythmias Hirosaki University Graduate School of Medicine Hirosaki Japan; ^3^ Cardiology, Cardiovascular Center Saiseikai Kumamoto Hospital Kumamoto Japan; ^4^ Department of Cardiology Institute of Medicine, University of Tsukuba Ibaraki Japan; ^5^ Department of Cardiology Kokura Memorial Hospital Fukuoka Japan; ^6^ Division of Cardiology, Cardiovascular Center Showa Medical University Koto Toyosu Hospital Tokyo Japan; ^7^ Department of Cardiovascular Medicine Toyohashi Heart Center Aichi Japan; ^8^ Department of Cardiology Tokyo Heart Rhythm Clinic Tokyo Japan; ^9^ Department of Cardiology Dokkyo Medical University Saitama Medical Center Saitama Japan; ^10^ Department of Cardiology Fujita Health University Toyoake Aichi Japan; ^11^ Department of Cardiovascular Medicine Japanese Red Cross Society Himeji Hospital Hyogo Japan; ^12^ School of Medical Technology Kurume University Fukuoka Japan; ^13^ Biostatistics Center Kurume University Fukuoka Japan

**Keywords:** atrial fibrillation, carina conduction gap, contact force, high‐power short‐duration, pulmonary vein isolation

## Abstract

**Background:**

High‐power short‐duration (HPSD) ablation is an established therapy for pulmonary vein (PV) isolation (PVI) in atrial fibrillation (AF), aiming to form efficient transmural lesions. Very HPSD (vHPSD) can further shorten ablation time but may increase the risk of acute PVI failure compared with HPSD. A combined HPSD and vHPSD strategy (90 W/50 W combination workflow) potentially balances efficiency and durability, though its clinical feasibility remains unknown. Therefore, this multicenter registry evaluated the acute and long‐term efficacy of a 90 W/50 W combination workflow for PVI in patients with paroxysmal AF.

**Methods:**

In this prospective study, a total of 101 consecutive patients with paroxysmal AF underwent PVI using radiofrequency ablation with a 90 W/50 W combination workflow. We evaluated acute outcomes, including first‐pass isolation and acute PV reconnection, and monitored atrial tachyarrhythmia recurrences over 12 months.

**Results:**

Median PVI procedure time was 35 min, with total procedure time at 105 min. First‐pass PVI was achieved in 58.4% of patients, including 74.3% in the right PV and 72.3% in the left PV. Acute PV reconnection occurred in 31.7% (32/101). In multivariate analysis, carina sites independently predicted acute PVI failure in both HPSD and vHPSD groups, while contact force also predicted failure in the HPSD group. After 1 year, 89.9% of patients remained free from documented atrial tachyarrhythmias.

**Conclusion:**

The 90 W/50 W combination workflow did not notably shorten procedure time or enhance first‐pass success. More standardized strategies, particularly in carina segments with higher contact force and ablation index under HPSD, may be required to ensure optimal lesion durability and favorable outcomes.

## Graphical abstract

This multicenter registry evaluated a 90 W/50 W combination workflow for pulmonary vein isolation (PVI) in 101 patients with paroxysmal atrial fibrillation. First‐pass PVI was achieved in 74.3% of right and 72.3% of left PVs. The 1‐year atrial tachyarrhythmia recurrence‐free survival was favorable at 89.9%, suggesting this strategy is a clinically effective option for achieving durable PVI.

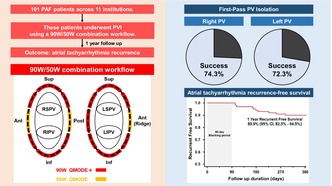

## 1 Introduction

Pulmonary vein (PV) isolation (PVI) for atrial fibrillation (AF) has become a widely accepted treatment [1]. Recently, PVI with radiofrequency ablation (RFA) has been performed employing a high‐power short‐duration (HPSD) strategy involving 40–50 W applied for 10–15 s. PVI with HPSD‐RFA has been reported to result in shorter ablation times, higher success rates for first‐pass isolation, and a lower recurrence rate of atrial arrhythmias at 12‐month follow‐up compared to low‐power long‐duration RFA [2, 3, 4]. In recent years, a very HPSD (vHPSD) strategy applying 90 W for 4 s with QDOT MICRO catheter (Biosense Webster Inc., Irvine, CA) has gained increasing popularity. The QDOT‐FAST Trial showed that all patients who underwent PVI with the vHPSD strategy achieved successful PVI, with 94.2% maintaining sinus rhythm 3 months after the procedure [5]. However, a previous report indicates that the vHPSD strategy, while tending to reduce the total ablation duration, has a lower first‐pass PVI success rate and a higher acute PV reconnection rate compared to the HPSD strategy [6]. The vHPSD setting may result in greater challenges in achieving transmural lesions compared to the HPSD setting. Previous animal research has indicated that the vHPSD setting produces shallower lesion depths compared to the HPSD setting [7, 8]. Additionally, our previous clinical study reported that left atrial (LA) wall thickness was associated with PV gaps after PVI performed with the vHPSD strategy [9].

We hypothesized that combining HPSD and vHPSD strategies could reduce procedure time while maintaining a high rate of first‐pass isolation. In this approach, an Ablation Index (AI) guided 50 W HPSD strategy was applied to areas presumed to have thicker LA walls, whereas the vHPSD strategy was used in areas with thinner wall thickness. This study aims to prospectively assess the acute and chronic feasibility of this combination workflow in a multicenter registry.

## 2 Methods

### 2.1 Study Population

This prospective multicenter registry study consecutively enrolled 101 patients with paroxysmal AF who underwent RFA using the 90 W/50 W combination workflow at 11 institutions across Japan. Patient enrollment was conducted between March and December 2023, with 1‐year follow‐up completed by December 2024. This study involving human participants was conducted in compliance with protocols approved by the institutional review board of Nihon University Itabashi Hospital and the institutional review boards of the participating hospitals (RK‐230110‐1).

### 2.2 Ablation Protocol

**FIGURE 1:**
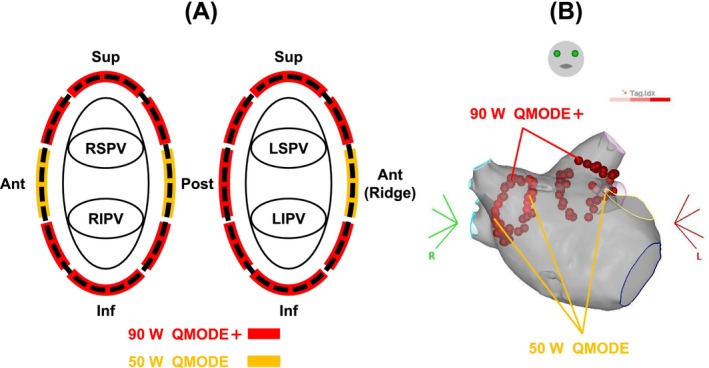
(A) 90 W/50 W combination workflow: HPSD was used in areas considered to have thicker LA wall, while very HPSD was applied in areas considered thinner. (B) Representative case: A procedure performed using the combination workflow. HPSD, high‐power short‐duration; LIPV, left inferior pulmonary vein; LSPV, left superior pulmonary vein; RIPV, right inferior pulmonary vein; RSPV, right superior pulmonary vein.

Heparin was administered during the procedure to target an activated clotting time in the range of 300–400 s. LA mapping was performed using the CARTO system (Biosense Webster). RFA was performed with a QDOT MICRO catheter, employing a combination of vHPSD and HPSD strategies (90 W/50 W combination workflow). In vHPSD, ablation was performed at 90 W for 4 s, whereas in HPSD, it was applied at 50 W with an AI of 400–550. In both settings, the target contact force was 10–15 g and the tag radius was 3 mm. The inter‐lesion distance was set to 4 mm, but in the posterior sites near the esophagus, the inter‐lesion distance was increased to 6 mm. Although the study protocol recommended esophageal monitoring, the specific method used was left to each institution's discretion. All patients underwent pre‐procedural cardiac computed tomography (CT) or magnetic resonance imaging (MRI) to assess PV and LA anatomy. The 90 W/50 W combination workflow (Figure 1) recommended a standardized approach: using the AI‐guided 50 W HPSD setting for areas with presumed thicker walls [10], such as the bilateral PV carinas, while using the 90 W vHPSD setting for other segments. However, the choice between vHPSD and HPSD for each PV segment was ultimately left to the operator's discretion, informed by the pre‐procedural CT or MRI imaging and intraprocedural findings.

First‐pass PVI success was defined as success in achieving PVI after the first round of ablation. After PVI, spontaneous PV reconnections were evaluated. As per the study protocol, administration of adenosine triphosphate (ATP) was recommended in all patients to assess dormant conduction. However, the total number of patients who actually received ATP was not recorded in the multicenter registry. Therefore, the proportion of dormant conduction relative to all ATP‐tested patients could not be calculated. Dormant conduction provoked by ATP was confirmed in 12 patients. Touch‐up ablation was performed on the conduction gaps until the PV gaps were eliminated. Carina ablation was not part of the initial strategy and was performed additionally only when deemed necessary by the operator. The endpoint of the procedure was defined as the successful completion of the PVI. The decision to perform additional procedures beyond PVI was left to the operator's discretion.

### 2.3 Collected Data

Among the collected data, the following were used for analysis: age, sex, body mass index, comorbidities (hypertension, diabetes mellitus, history of stroke, heart failure, or ischemic heart disease), oral anticoagulant type, type of direct oral anticoagulant used, antiarrhythmic drug, hemoglobin concentration, creatinine and creatinine clearance at the time of registry enrollment, transthoracic echocardiography‐derived LA diameter, and left ventricular ejection fraction. We collected data on the detailed ablation parameters including the procedure time, fluoroscopy time, right and left wide‐area circumferential ablation perimeters (cm).

Ablation tag data recorded in the CARTO system were analyzed in 80 patients. Tags around acute PVI failure sites, including first‐pass PVI failure sites and acute reconnection sites, were classified as non‐durable tags, whereas all other tags were classified as durable tags. Ablation tag locations and gap sites were categorized into the following nine segments for both right PV (RPV)–PVI and left PV (LPV)–PVI analyses: superior, superior‐anterior, carina‐anterior, inferior‐anterior, inferior, inferior‐posterior, carina‐posterior, superior‐posterior, and carina. The carina region was defined as comprising three segments: carina‐anterior, carina, and carina‐posterior. The following operator‐adjustable parameters were evaluated: Max power (W) per point, ablation time per point (sec), Contact force (g), Impedance drop (Ω), AI for the 50 W setting, ablation lesion segment (carina region or non‐carina segment).

### 2.4 Follow‐Up and Study Endpoints

The follow‐up began on the day of the initial ablation performed during the enrollment period at each center. All patients were followed up at the hospital outpatient clinic. Follow‐up was conducted at 3, 6, and 12 months post‐ablation to assess atrial tachyarrhythmia recurrence using a 12‐lead electrocardiogram (ECG), Holter ECG, and an ECG event recorder when necessary. A 9‐month follow‐up was performed optionally. A 90‐day blanking period was applied following PVI to exclude early recurrences. Any documented episode of atrial tachyarrhythmia lasting longer than 30 s was considered a recurrence.

### 2.5 Statistical Analysis

Continuous variables are expressed as the mean ± standard deviation (SD) or as the median with interquartile range (IQR; 25th to 75th percentile). Categorical variables are presented as counts and percentages. Continuous variables were compared using Student's *t*‐test or the Mann–Whitney *U* test, as appropriate. Categorical variables were analyzed using the chi‐square test, except when the expected cell count was < 5, in which case Fisher's exact test was applied. A multivariate logistic regression analysis was performed to identify factors associated with acute PVI failure sites. Variables that were significant in the univariate analysis were included in the model. Receiver operating characteristic (ROC) curves were plotted to determine the cutoff value of contact force for acute PVI failure. Kaplan–Meier analysis was performed to evaluate atrial tachyarrhythmia recurrence‐free survival. All statistical analyses were performed using JMP Pro 17 software (SAS Institute, Cary, NC), except for the comparison of acute PVI failure incidence among the 11 institutions, which was evaluated using Fisher's exact test with SAS 9.4 (SAS Institute, Cary, NC). A *p*‐value of ≦ 0.05 was considered statistically significant.

## 3 Results

### 3.1 Patient and Procedural Characteristics

Patient characteristics are shown in Table 1. The patients in this study had a mean age of 65 ± 12 years, and 67 (66.3%) patients were male. The LA diameter and left ventricular ejection fraction were 37.6 ± 5.2 mm and 64.4% ± 6.0%, respectively. As shown in Table 1, esophageal temperature monitoring using an esophageal temperature probe was performed in 64 patients, among whom maximal esophageal temperature was documented in 56. The mean maximal temperature in these patients was 40.8°C ± 1.8°C. All patients received anticoagulation therapy during the periprocedural period. 66 (65.3%) patients were also on antiarrhythmic drugs before PVI. Among the patients, 44 were classified into the Non‐PV Gap group, characterized by the absence of both first‐pass PVI failure and acute reconnection. In contrast, 57 patients were categorized into the PV Gap group, defined by the presence of either or both conditions. Although the body mass index tended to be higher in the PV Gap group, the difference was not statistically significant. No other patient characteristics differed significantly between the two groups.

**TABLE 1:** Patient characteristics.

	Total (*n* = 101)	PV gap group (*n* = 57)	Non‐PV gap group (*n* = 44)	*p*
Clinical characteristics				
Age (years)	65 ± 12	64 ± 13	67 ± 12	0.393
Male sex	67 (66.3)	38 (66.7)	29 (65.9)	0.936
Body mass index (kg/m^2^)	23.8 ± 3.8	24.3 ± 4.0	23.2 ± 3.4	0.124
Hypertension	53 (52.5)	28 (49.1)	25 (56.8)	0.443
Diabetes mellitus	18 (17.8)	11 (19.3)	7 (15.9)	0.659
Heart failure	6 (5.9)	4 (7.0)	2 (4.6)	0.694
History of stroke	6 (5.9)	4 (7.0)	2 (4.6)	0.694
Ischemic heart disease	7 (6.9)	2 (3.5)	5 (11.4)	0.235
Laboratory variables				
Hemoglobin (g/dL)	13.9 ± 1.5	14.0 ± 1.5	13.8 ± 1.6	0.405
Creatinine (mg/dL)	0.81 ± 0.20	0.82 ± 0.17	0.80 ± 0.24	0.616
Creatinine clearance (mL/min)	82.4 ± 28.0	84.5 ± 26.9	79.7 ± 29.4	0.394
Echocardiographic measurements				
Left ventricular ejection fraction (%)	64.4 ± 6.0	64.0 ± 5.1	65.0 ± 7.0	0.407
Left atrial diameter (mm)	37.6 ± 5.2	37.6 ± 5.4	37.7 ± 4.8	0.916
Medications				
Oral anticoagulants during periprocedural period	101 (100)	57 (100)	44 (100)	—
Direct oral anticoagulants	99 (98)	56 (98)	43 (98)	—
Warfarin	2 (2)	1 (2)	1 (2)	—
Antiarrhythmic drugs before PVI				
Class I–IV	66 (65.3)	41 (71.9)	25 (56.8)	0.114
Class I or III or bepridil	34 (33.7)	19 (33.3)	15 (34.1)	0.936
Esophageal monitoring				
Esophageal temperature probe	45 (44.6)	—	—	—
Esophagography	9 (8.9)	—	—	—
Intracardiac echocardiography	4 (4.0)	—	—	—
Esophageal temperature probe and esophagography	19 (18.8)	—	—	—
None	24 (23.8)	—	—	—
Ablation‐related characteristics				
Additional procedures other than PVI	52 (51.5)	31 (54.4)	21 (47.7)	0.507
Left atrial linear ablation	5 (5.0)	2 (3.5)	3 (6.8)	0.651
Electrogram‐based ablation	2 (2.0)	1 (1.8)	1 (2.3)	1.00
SVC isolation	27 (26.7)	17 (29.8)	10 (22.7)	0.424
Non‐PV trigger ablation	4 (4.0)	3 (5.3)	1 (2.3)	0.630
Cavo tricuspid isthmus ablation	22 (21.8)	13 (22.8)	9 (20.5)	0.776
Right wide‐area circumferential ablation perimeters (cm)^a^	11.1 (9.9, 12.6)	11.1 (9.9, 12.9)	11.1 (9.9, 11.7)	0.515
Left wide‐area circumferential ablation perimeters (cm)^a^	11.7 (10.2, 13.0)	11.7 (9.9, 13.2)	11.6 (10.2, 12.2)	0.619
Total fluoroscopic time (min)	9 (5.3, 19)	10.2 (6.3, 20.5)	7 (5, 16.2)	0.110
PVI procedure time (min)	35 (26, 46.5)	40 (30, 56)	29.5 (23, 37)	0.0001
Total procedure time (min)	105 (85.5, 132)	114 (100, 155)	90 (81.5, 111)	< 0.0001

*Note:* Values are shown as the mean ± SD, median (25th, 75th interquartile range) or *n* (%). Percentages may not total 100 due to rounding.

Abbreviations: BMI, body mass index; LA, left atrium; LAD, left atrial dimension; LVEF, left ventricular ejection fraction; PV, pulmonary vein; PVI, pulmonary vein isolation; SVC, superior vena cava; WACA, wide‐area circumferential ablation.

^a^ Analysis was performed on 100 patients due to one missing data point.

Procedural characteristics are presented in Table 1. A total of 52 patients underwent additional procedures other than PVI. Among them, the common procedures were Superior Vena Cava isolation (26.7%) and Cavo tricuspid isthmus ablation (21.8%). The median PVI procedure time was 35 min, and the median total procedure time was 105 min. Despite similar wide‐area circumferential ablation lengths in both the RPV and LPV and no difference in fluoroscopic time, the PVI procedure time and total procedure time were significantly longer in the PV Gap group compared to the Non‐PV Gap group.

### 3.2 Effective and Safety Outcome

**FIGURE 2:**
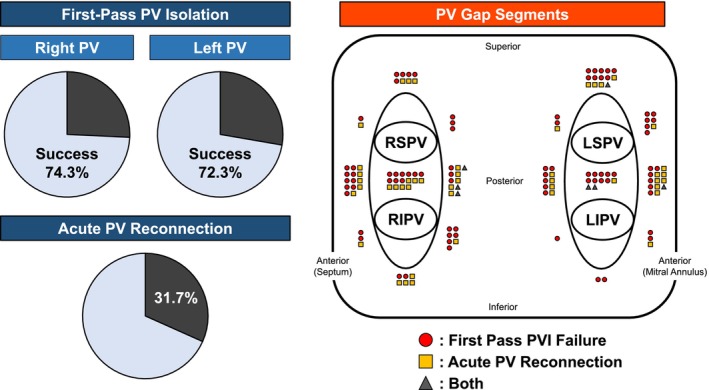
Left: First‐pass pulmonary vein (PV) isolation rate and acute PV reconnection rate. Right: Regional distribution of PV gaps at the junction between the PVs and the left atrium surrounding each PVI line. LIPV, left inferior pulmonary vein; LSPV, left superior pulmonary vein; PV, pulmonary vein; PVI, pulmonary vein isolation; RIPV, right inferior pulmonary vein; RSPV, right superior pulmonary vein.

**FIGURE 3:**
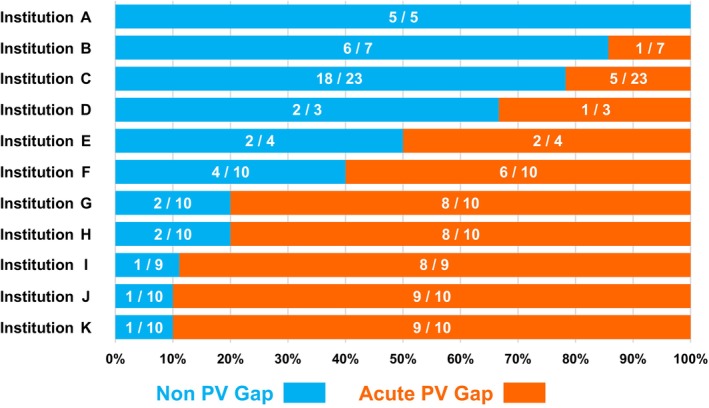
Proportion of acute PV gap groups and nonPV gap groups at each institution. Blue bars indicate nonPV gap groups, and orange bars indicate acute PV gap groups. White numbers inside the bars indicate cases/total cases treated at each institution. PV, pulmonary vein.

At the end of the procedure, complete PVI was successfully achieved in all patients. First‐pass PVI success was achieved in 59 of 101 patients (58.4%) (Table 2). First‐pass PVI was achieved in 75 of 101 patients (74.3%) for the right PV and in 73 of 101 patients (72.3%) for the left PV. Acute PV reconnection during the procedure was observed in 32 patients (31.7%) (Figure 2). The distribution of first‐pass PVI failure sites and acute PV reconnection sites across the segments is shown in Figure 2. First‐pass PVI failure was observed in 90 segments. Among these, 48 segments (53.3%) were located in the carina region. Acute PV reconnections, including spontaneous reconnections and dormant conduction, were observed in 53 segments, of which 35 segments (66.0%) were located in the carina region. The proportions of patients in the PV gap and non‐PV gap groups at each institution are shown in Figure 3. There were significant differences in the incidence of acute PV gaps among the 11 institutions (*p* < 0.0001, Figure 3).

**TABLE 2:** Effective and safety outcome.

	Total (*N* = 101)
Acute effective outcome	
First‐pass PVI success	59 (58.4)
First‐pass PVI success in right PV	75 (74.3)
First‐pass PVI success in left PV	73 (72.3)
Acute PV reconnection during procedure	32 (31.7)
Ablation related complications	
Cardiac tamponade	1 (1.0)
Dry cough after procedure	1 (1.0)

*Note:* Values are shown as the *n* (%).

Abbreviations: PV, pulmonary vein; PVI, pulmonary vein isolation.

Ablation‐related complications were shown in Table 2. One case of cardiac tamponade occurred during the procedure, and one patient developed postoperative dry cough.

### 3.3 Ablation Related Parameters

Ablation tag parameters are summarized in Table 3. The AI for the 50 W tags in our study was 454 ± 51. The multivariate logistic regression analysis was conducted separately for 50 W and 90 W ablation tags to evaluate factors associated with non‐durable tags. For 50 W ablation tags, after adjusting for ablation duration, contact force, and carina lesion in the model, the analysis revealed that contact force (Odds ratio (OR) 0.95, 95% confidence interval (95% CI) 0.92–0.98, *p* = 0.003) and carina lesion (OR 4.50, 95% CI 2.87–7.04, *p* < 0.001) were significantly associated with non‐durable tags. An OR of 0.95 for contact force suggests that higher contact force is associated with an increased likelihood of achieving durable lesion formation (i.e., a decreased likelihood of non‐durable tags). Additionally, ROC curve analysis stratified by carina versus non‐carina lesions was performed to identify a cutoff value for contact force. Among carina segments, the cutoff value was 12.7, with an area under the curve (AUC) of 0.56. In contrast, for 90 W ablation tags, after adjusting for contact force, impedance drop, and carina lesion in the model, the analysis showed that impedance drop (OR 0.91, 95% CI 0.86–0.96, *p* = 0.001) and carina lesion (OR 2.56, 95% CI 1.78–3.68, *p* < 0.001) were significantly associated with non‐durable tags.

**TABLE 3:** Ablation tag parameters and odds ratio for non‐durable tags.

Variable	50 W tags			Univariable analysis		Multivariable analysis	
	All tags (*N* = 2030)	Durable tags (*N* = 1848)	Non‐durable tags (*N* = 182)	OR (95% CI)	*p*	OR (95% CI)	*p*
Max power (W)	49.8 ± 1.7	49.8 ± 1.8	49.9 ± 1.5	1.02 (0.92–1.12)	0.733		
Ablation duration (s)	14.6 ± 4.3	14.5 ± 4.3	15.2 ± 4.9	1.03 (1.00–1.07)	0.050	1.01 (0.97–1.05)	0.657
Contact force (g)	12.3 ± 5.6	12.4 ± 5.7	11.1 ± 4.1	0.95 (0.92–0.98)	0.002	0.95 (0.92–0.98)	0.003
Impedance drop (Ω)	7.3 ± 3.6	7.3 ± 3.7	7.0 ± 3.1	0.98 (0.94–1.02)	0.272		
Ablation index	454 ± 51	454 ± 51	452 ± 55	1.00 (1.00–1.00)	0.563		
Carina lesions	1287 (63.4)	1128 (61.0)	159 (87.4)	4.41 (2.82–6.90)	< 0.0001	4.50 (2.87–7.04)	< 0.0001

Variable	90 W Tags			Univariable analysis		Multivariable analysis	
	All tags (*N* = 3593)	Durable tags (*N* = 3431)	Non‐durable tags (*N* = 162)	OR (95% CI)	*p*	OR (95% CI)	*p*
Max power (W)	89.9 ± 3.0	89.9 ± 2.9	89.8 ± 3.9	0.99 (0.95–1.03)	0.664		
Ablation duration (s)	3.9 ± 0.4	3.9 ± 0.4	3.9 ± 0.4	1.07 (0.70–1.64)	0.739		
Contact force (g)	15.8 ± 7.6	15.8 ± 7.6	14.6 ± 5.1	0.98 (0.95–1.00)	0.046	0.98 (0.96–1.00)	0.105
Impedance drop (Ω)	8.4 ± 3.4	8.4 ± 3.3	7.3 ± 3.4	0.89 (0.85–0.94)	< 0.0001	0.91 (0.86–0.96)	0.001
Carina lesions	459 (12.8)	414 (12.1)	45 (27.8)	2.80 (1.96–4.01)	< 0.0001	2.56 (1.78–3.68)	< 0.0001

*Note:* Values are shown as the mean ± SD or *n* (%).

Abbreviations: CI, confidence interval; OR, odds ratio.

### 3.4 Follow‐Up

During a median follow‐up period of 372 days (IQR: 365–387), three patients were lost to follow‐up. During follow‐up, major clinical events included one case of intracranial hemorrhage under oral anticoagulation therapy, one case of diverticular bleeding (which led to the discontinuation of anticoagulation therapy), one death due to sepsis, and one case of pacemaker implantation for sick sinus syndrome. During the follow‐up period, no patients presented with symptoms suggestive of a significant esophageal injury. At 1 year, oral anticoagulants were continued in 55.1% of patients.

**FIGURE 4:**
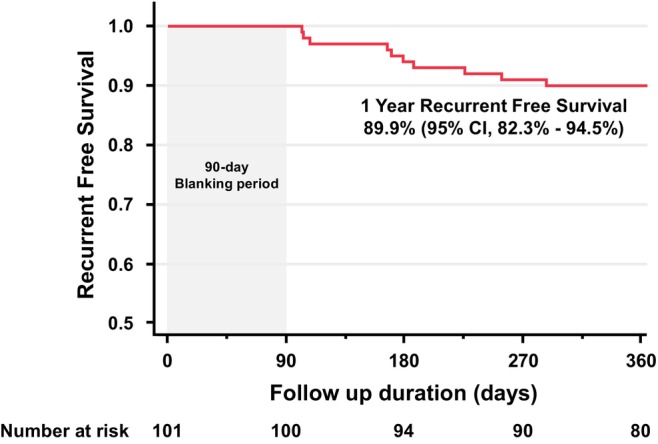
Kaplan–Meier analysis of atrial tachyarrhythmia recurrence‐free survival.

Atrial tachyarrhythmia recurrence was observed in 10 patients during this period. The recurrence patterns included AF in eight patients and atrial tachycardia (including atrial flutter) in two patients. There was no significant difference in atrial tachyarrhythmia recurrence between the PV gap group (5 of 57 patients, 8.8%) and the non‐PV gap group (5 of 44 patients, 11.4%) (*p* = 0.744). A repeat catheter ablation procedure was performed in five patients who experienced atrial tachyarrhythmia recurrence, and PV reconnection was observed in two of these cases. With 92.8% of patients off antiarrhythmic drugs (defined as no use of Class I, Class III agents, or bepridil at 1 year), the single‐procedure, one‐year event‐free survival rate was 89.9% (95% CI, 82.3%–94.5%; Figure 4).

## 4 Discussion

### 4.1 Major Findings

This study has four major findings: (1) The median PVI procedure time of the 90 W/50 W combination workflow was 35 min, and the median total procedure time was 105 min. (2) First‐pass PVI success of the 90 W/50 W combination workflow was achieved in 59 of 101 patients (58.4%), including 75 patients (74.3%) for the right PV and 73 patients (72.3%) for the left PV. Acute PV reconnection during the procedure was observed in 32 patients (31.7%). (3) Multivariate logistic regression analysis identified carina sites as an independent predictor of acute PVI failure in both HPSD and vHPSD. In addition, contact force was also identified as an independent predictor of acute PVI failure in the HPSD group. (4) The 1‐year atrial tachyarrhythmia recurrence‐free survival rate was 89.9%.

### 4.2 PVI Procedure Time

Previous reports have consistently demonstrated shorter procedure times with vHPSD compared to HPSD [6, 11, 12]. In our study, the total procedure time was relatively long, with a median of 105 min, even compared to previous reports on HPSD [6, 11, 12]. This discrepancy may be partly due to differences in the definition of procedure time across studies. In our study, total procedure time was defined as the interval from femoral puncture to sheath removal, and 51.5% of patients underwent additional procedures other than PVI. These factors may limit the comparability of our results with those of prior studies. In contrast, for PVI procedure time, a previous study by Nándor Szegedi et al. used the same definition as ours and reported a median PVI procedure time of 27 min (IQR 24–33) with vHPSD and 34 min (IQR 30–44) with HPSD [13]. In our study, the median PVI procedure time was 35 min, which is comparable to that of the HPSD in their report. Notably, when limited to the non‐PV gap group, the median time decreased to 29.5 min, approaching the values reported for vHPSD. These findings suggest that reducing PV gaps may contribute to shorter procedure times and underscore the need for a more standardized and refined ablation strategy.

### 4.3 First‐Pass PVI Success and Chronic Outcome

Previous reports have shown that the first‐pass PVI success rate with HPSD ranges from 79% to 96.6% [4, 14], whereas the rate with vHPSD has been reported to range between 75% and 83.9% [9, 12]. In the present study, in which a 90 W/50 W combination workflow was applied, the first‐pass PVI success rate was 74.3% for the right PVI and 72.3% for the left PVI, which is comparable to or slightly lower than rates reported in previous studies. A significant contributor to this result was the challenge of ablating the carina region.

O'Neill et al. reported that acute PV gaps often clustered at the carina [12]. Similarly, our study showed that carina lesions at the carina and lower contact force during HPSD ablation were independently associated with non‐durable tags. The tendency for non‐durable lesion formation at the carina is consistent with previous reports [15, 16], and these results suggest that achieving durable lesions at the carina remains challenging. It has been reported that in the HPSD strategy using QMODE, tissue temperature at the ablation site is lower due to the cooling effect of irrigation [7]. Therefore, applying greater contact force may theoretically enhance energy delivery and increase tissue temperature, potentially resulting in more effective lesion formation. However, the AUC of the ROC analysis for contact force was 0.56 in carina segments in this study. Thus, although contact force was statistically significant, its predictive value for lesion durability appears limited compared to the impact of lesion at the carina. This may be partly explained by the presence of epicardial conduction at the carina, which can render even high‐contact‐force lesions ineffective. Epicardial pathways—such as connections between the right‐sided carina and right atrium, or between the left‐sided carina and the ligament of Marshall—can bypass endocardial ablation lines and contribute to lesion non‐durability in this region [17, 18].

Furthermore, another key finding was the inter‐institutional variability inherent in this multicenter study. A previous study suggested that operator volume can influence the first‐pass PVI success rate [15]. In fact, in the present study, there were significant differences in the incidence of acute PV gaps among the 11 institutions (*p* < 0.0001, Figure 3). Beyond general operator experience, this variability may also reflect different applications of the study protocol. For instance, the discretion in choosing between the 50 W and 90 W settings could have led to different strategies across centers. Particularly regarding the AI, the target AI for 50 W applications had a wide range (400–550). While the mean AI in our study was 454 ± 51, institutional preferences for targets on the lower end of this spectrum could be a key factor, as this may be insufficient for creating durable lesions at the carina [16].

In the present study, despite a relatively low first‐pass PVI rate of approximately 70%, the one‐year event‐free survival rate was favorable at 89.9%. Although many previous studies have reported an association between higher first‐pass PVI rates and lower long‐term recurrence rates [19, 20], our findings revealed a discrepancy between the acute and long‐term outcomes. This may be attributed to the thorough performance of additional touch‐up ablation to eliminate residual gaps. Ensuring complete gap elimination may contribute to favorable long‐term outcomes, even when first‐pass PVI is not achieved. Notably, this outcome was achieved with 92.8% of patients off antiarrhythmic drugs at 1 year, and procedure‐related complications were minimal, with only one case (1%) of cardiac tamponade and one case of post‐procedural dry cough (Table 2). These results suggest that vHPSD/HPSD using the QDOT MICRO catheter offers a safety profile comparable to pulsed field ablation, with a 1‐year arrhythmia‐free rate that may match or exceed those outcomes reported in recent pulsed field ablation trials, such as the MANIFEST‐PF Registry and the AdmIRE Pivotal Trial, which showed rates of 81.6% and 75.4% for paroxysmal AF, respectively [21, 22]. Taken together, these findings indicate that RFA, particularly with the 90 W/50 W combination workflow, remains a competitive and effective treatment option for AF in contemporary clinical practice.

### 4.4 Study Limitations

This study has several limitations. First, tags obtained from the same patient were treated as independent samples in this study. This may introduce bias, as within‐patient correlations were not accounted for. Second, inter‐lesion distance and bipolar voltage could not be assessed in this study. Previous reports have shown that inter‐lesion distance and bipolar voltage were associated with acute PVI gap [16, 23, 24], which may have influenced the present results. Third, the absence of routine post‐procedural endoscopy precluded assessment of the incidence of subclinical esophageal thermal injury. Finally, our statistical analysis did not adjust for center effects or within‐patient clustering, which is another limitation of this study.

## 5 Conclusion

In this study, the 90 W/50 W combination workflow, in which HPSD and vHPSD were simply applied based on presumed wall thickness, did not result in a shorter procedure time or a higher first‐pass PVI success rate. To further enhance procedural efficacy, a more standardized and optimized ablation strategy may be required. Specifically, in carina segments, targeting a higher contact force and ablation index during HPSD may be necessary to ensure durable lesion formation and improve procedural outcomes.

## Disclosure

Registry and the registration no. of the study/trial: This study was registered in the UMIN Clinical Trials Registry (UMIN‐CTR, UMIN000050344; registered on 15 February 2023).

## Ethics Statement

The research protocol was reviewed and approved by the institutional review board of Nihon University Itabashi Hospital and the institutional review boards of the participating hospitals (RK‐230110‐1).

## Consent

Informed consent was obtained from all participants in the study.

## Conflicts of Interest

Dr. Okumura has received speaker honoraria from AstraZeneca K.K. and Johnson & Johnson K.K.; research funding from MEDTRONIC JAPAN CO. LTD., MicroPort CRM Japan, and Bayer Healthcare; and endowed chair support from Abbott Japan LLC, Boston Scientific Japan K.K., MEDTRONIC JAPAN CO. LTD., Japan Lifeline Co. Ltd., and BIOTRONIK. Dr. Nagashima has received speaker honoraria from Daiichi‐Sankyo, Johnson & Johnson/Biosense Webster, Medtronic Japan, Boston Scientific Japan, and Abbott Medical Japan. Dr. Kimura is affiliated with an endowed department supported by Medtronic Japan Co. Ltd., Fukuda Denshi Co. Ltd., and Japan Lifeline Co. Ltd. Dr. Kimura has received honoraria for lectures or speaker engagements from Medtronic Japan Co. Ltd., Boston Scientific Japan K.K., Abbott Medical Japan LLC, Toray Industries Inc. and Johnson & Johnson K.K. Dr. Komatsu has received honoraria from Abbott and Johnson & Johnson. Dr. Kuwahara has received speaker honoraria from Johnson & Johnson and Abbott. Dr. Harada has received speaker fees from Medtronic and Abbott. No other authors have any conflicts of interest to disclose.

## Data Availability

The data supporting the findings of this study are not available.
